# A Case of Latent Syphilis in Early Pregnancy: An Incidental Finding at Seven Weeks Gestation

**DOI:** 10.7759/cureus.76627

**Published:** 2024-12-30

**Authors:** Shahd Abouelenen, Kiran Kumar

**Affiliations:** 1 College of Medicine, Gulf Medical University, Ajman, ARE; 2 Internal Medicine, Thumbay University Hospital, Ajman, ARE

**Keywords:** hypertensive disorders of pregnancy, latent syphilis, syphilis in pregnancy, treatment failure, treponema pallidum

## Abstract

*Treponema pallidum*, the bacteria that causes syphilis, is typically acquired through sexual contact but can also be transmitted transplacentally (through the placenta), causing congenital infection. Syphilis in pregnancy is a major contributing factor to perinatal morbidity and mortality. Untreated neonates may develop complications affecting the central nervous system, bones, joints, teeth, eyes, and skin. This report highlights the incidental discovery of syphilis in an asymptomatic 22-year-old primigravida (pregnant for the first time) at seven weeks gestation through a positive *Treponema pallidum* hemagglutination assay and treponemal antibody test during routine antenatal screening, while testing negative for human immunodeficiency virus (HIV). A rapid plasma reagin (RPR) test confirmed the infection with a titer of 1:32, though her partner tested negative for syphilis and HIV. She had a genital rash five months ago treated by a gynecologist in Thailand and remained asymptomatic. The patient was managed with benzathine penicillin G 2.4 million units intramuscularly once weekly for three weeks. Three months post-treatment, the RPR titer decreased to 1:16; however, the patient developed pre-eclampsia and required a cesarean delivery at 30 weeks and five days. Before delivery, the titer increased to 1:32. To prevent congenital syphilis, she received one prenatal and two postnatal benzathine penicillin G injections. The newborn was born without deformities, as confirmed by comprehensive evaluation, and follow-up revealed a successful reduction of the RPR titer to 1:8 three months postpartum. This case underscores the importance of routine antenatal screening for early syphilis detection and treatment, even in asymptomatic patients, to prevent maternofetal complications.

## Introduction

Syphilis, caused by *Treponema pallidum*, is a systemic sexually transmitted infection that can be transmitted transplacentally to the fetus during pregnancy, leading to severe fetal complications and perinatal mortality. Congenital syphilis can result in serious complications such as meningitis, mucocutaneous lesions, hemolytic anemia, hepatosplenomegaly, jaundice, blindness, and deafness due to nerve damage [1]. Antenatal screening and early treatment are crucial for preventing these outcomes.

According to the latest guidelines by the Centers for Disease Control and Prevention (CDC), serologic screening for syphilis is recommended for all pregnant women during their first prenatal care visit [2]. Moreover, for pregnant women who reside in communities with high syphilis prevalence and those at risk of contracting syphilis during pregnancy, serologic testing should be repeated twice during the third trimester at 28 weeks gestation and again at delivery [2]. Pregnant women who test positive on treponemal tests, such as *Treponema pallidum* passive particle agglutination assay, enzyme immunoassay, chemiluminescent immunoassay, or immunoblot, should undergo additional quantitative nontreponemal testing with Venereal Disease Research Laboratory or rapid plasma reagin (RPR) test [2]. This additional testing is crucial for establishing baseline titer levels, monitoring the effectiveness of treatment, and tracking disease progression or potential reinfection. In addition, human immunodeficiency virus (HIV) testing should be offered to all women diagnosed with syphilis to screen for potential coinfection [2].

Benzathine penicillin G is the known effective treatment for syphilis during pregnancy [3]. However, pregnant women with penicillin allergy should undergo immediate desensitization and, subsequently, be treated with penicillin G [3]. This report details a unique asymptomatic case of syphilis detected during first-trimester antenatal screening.

## Case presentation

A 22-year-old primigravida at seven weeks gestation incidentally tested positive for both *Treponema pallidum* hemagglutination assay (TPHA) and treponemal antibody-qualitative test during routine antenatal screening, while testing negative for HIV through enzyme-linked immunosorbent assay (ELISA). Clinical examination showed no apparent ulcers or lesions in the genital area and no rash in any part of the body. Systemic review was unremarkable and the patient did not have any signs or symptoms indicating an active syphilis infection. However, the patient reported having a genital rash approximately five months ago which was treated by a gynecologist in Thailand. She remained asymptomatic following the treatment. She was unmarried and sexually active with one partner. Her partner tested negative for syphilis and HIV.

The patient was counseled extensively and advised to do an RPR titer to assess the severity of the infection. The test came back reactive with a titer of 1:32, confirming the syphilis infection. The patient was informed about the potential complications of untreated syphilis during pregnancy as well as the neonatal adverse outcomes of congenital syphilis. The significance of early detection and treatment was also highlighted to protect the health and well-being of both the mother and fetus.

Since the exact duration of infection remains unknown due to the lack of clinical symptoms, the World Health Organization (WHO) Guideline on Syphilis Screening and Treatment for Pregnant Women recommends treatment with benzathine penicillin G 2.4 million units intramuscularly once weekly for three consecutive weeks. The patient did not have a history of penicillin allergy. To confirm this, a test dose of 0.1 mL intradermally was administered and the patient did not show signs of allergy. After confirming the absence of penicillin allergy, the patient started the treatment plan. Intramuscular injection of 1.2 million units of benzathine penicillin G was administered in each gluteal area weekly. Following the injection, the patient was monitored for 30 minutes. The treatment was completed successfully without any complications, and the patient was advised to repeat the RPR titer every three months, as per the WHO guidelines, to assess the effectiveness of the treatment.

Three months after the initial therapy, the patient (19 weeks gestation) showed a twofold decrease in the RPR titer to 1:16. Ultrasound assessments were done to evaluate fetal growth, amniotic fluid levels, and placental function. There were no signs of intrauterine growth restriction or other abnormalities. At 30 weeks and five days, she developed preeclampsia, characterized by headache, peripheral edema, mild proteinuria (+1), and severe hypertension of 160/180 mmHg on two occasions. Despite receiving antihypertensive medication, her blood pressure remained uncontrolled, and signs of fetal distress were detected. She was started on magnesium sulfate for seizure prophylaxis, and corticosteroids were administered to promote fetal lung maturity. Due to concerns for both maternal and fetal health, an emergency cesarean section was performed.

Before delivery, the titer was repeated and had increased back to 1:32. To prevent congenital syphilis, the patient was administered one prenatal and two postnatal benzathine penicillin G injections. The child was born weighing 1.09 kg and underwent a comprehensive evaluation to rule out congenital syphilis. Tests included an RPR test, skeletal survey, ophthalmic examination, newborn hearing screening, and complete blood count. All tests were negative, confirming the absence of congenital syphilis. The mother’s blood pressure was monitored postpartum and stabilized at 120/80 mmHg. Repeat RPR titer three months postpartum was 1:8, achieving a successful fourfold titer reduction. The details of the patient’s test results are summarized in Table 1.

**Table 1:** Patient’s test results. ELISA: enzyme-linked immunosorbent assay; TPHA: *Treponema pallidum* hemagglutination assay; RPR: rapid plasma reagin

Test	Result	Reference range
ELISA	Nonreactive	Nonreactive
TPHA	Positive	Negative
Treponemal antibody-qualitative test	Positive	Negative
RPR (initial at seven weeks)	Reactive 1:32	Nonreactive
RPR (three months after initial treatment at 19 weeks gestation)	Reactive 1:16	Nonreactive
RPR (before delivery at 30 weeks and five days)	Reactive 1:32	Nonreactive
RPR (three months postpartum)	Reactive 1:8	Nonreactive

## Discussion

Syphilis is a curable sexually transmitted infection caused by the bacterium *Treponema pallidum* and can result in significant morbidity and mortality. Transmission occurs through sexual contact with infectious lesions, blood transfusion, or from mother to child (vertical transmission). In cases of vertical transmission that remain undiagnosed or untreated early, the fetus suffers devastating health consequences. Depending on the stage of syphilis, adverse birth outcomes (ABOs) are seen in approximately 50-80% of maternal syphilis cases when untreated, treated late, or not treated with penicillin [4]. Additionally, stillbirth (most common), neonatal death, prematurity, low birth weight, and congenitally infected infants are among the severe ABOs [4].

In 2022, the WHO reported 700,000 cases of congenital syphilis worldwide. These cases of maternal syphilis were associated with around 150,000 early fetal deaths and stillbirths, 70,000 neonatal deaths, 55,000 preterm or low birth weight births, and 115,000 infants diagnosed with congenital syphilis [5]. Moreover, syphilis is divided into four stages: primary, secondary, latent, and tertiary. When primary or secondary syphilis is left untreated yet its clinical symptoms resolve, latent syphilis develops [1]. Although our patient was likely in the latent stage due to the lack of symptoms at diagnosis, the prior genital rash suggests the likelihood of primary or secondary syphilis history. WHO guidelines recommend treating late latent syphilis (>1 year), latent syphilis of unknown duration, and tertiary syphilis with 7.2 million units total of benzathine penicillin G, administered as three doses of 2.4 million units intramuscularly each at one-week intervals [6].

The partner’s negative syphilis test result, despite recent sexual contact, does not exclude the possibility of infection due to several reasons. First, syphilis has a window period of two to three weeks during which serologic tests may give false-negative results. Additionally, the absence of symptoms or lesions in the partner at the time of testing may decrease the likelihood of accurate detection. It is important to highlight that the patient’s diagnosis was confirmed by both treponemal and nontreponemal tests, which provide high diagnostic sensitivity and specificity, effectively confirming the syphilis infection in the patient regardless of the partner’s test result. This underscores the significance of routine antenatal screening, independent of partner testing.

Our patient’s case is unique, as it highlights two noteworthy learning points: treatment failure and an observed association between syphilis and hypertensive disorders of pregnancy (HDP). The absence of a reduction in RPR titer despite receiving the standard three-dose benzathine penicillin G treatment indicates possible treatment failure. In such cases, the CDC recommends an additional treatment course of three weekly doses of 2.4 million units [3]. Moreover, in a comparable case documented in Indonesia [7], a pregnant patient with latent syphilis also presented with a high initial RPR titer (1:32) and required extended serologic monitoring before achieving a fourfold titer reduction over six months. Given the possibility of treatment failure, this finding supports the need for persistent monitoring.

The occurrence of pre-eclampsia in both cases highlights the potential association between syphilis and HDP, though it is not yet well established. It is supported by recent research conducted in China, which observed that the rate of HDP was notably higher among pregnant women with syphilis (12.3%) compared to the control group (3.8%), indicating a significant association [8]. Another study done in the United States and published in the American Journal of Obstetrics and Gynecology reported an increased risk of late-onset hypertensive disorders, up to 19% in women exposed to *Treponema pallidum* during pregnancy [9]. These findings emphasize the need for vigilant follow-up and timely intervention in managing syphilis during pregnancy. However, it is important to acknowledge the limitations of this case report, including the lack of detailed information on the treatment administered in Thailand and the absence of partner follow-up details.

## Conclusions

This case report highlights the complexities of managing syphilis in pregnancy, especially in asymptomatic cases. The fluctuating RPR titers despite standard therapy highlight the potential for treatment failure and the need for extended monitoring. Moreover, it points out a possible association between syphilis and HDP, an emerging area of concern that encourages further research. Untreated syphilis in pregnancy can result in severe adverse birth outcomes, including miscarriage, stillbirth, preterm delivery, and congenital syphilis. In addition, the prevention strategies should encourage routine antenatal screening for syphilis in the first trimester, especially in high-risk populations. Public health initiatives should include more awareness campaigns about syphilis, its potential complications, and the importance of timely diagnosis and treatment. This case serves as a reminder of the significance of routine antenatal screening for the early detection and treatment of syphilis in pregnancy to prevent maternal and fetal complications.
